# Biosecurity and Diagnosis of Viral Hemorrhagic Fevers: Strategic Considerations for Military Medicine

**DOI:** 10.3390/diagnostics16131968

**Published:** 2026-06-24

**Authors:** Salvatore Giovanni De-Simone, Andreia Carneiro da Silva, Marianne Melo Monnerat, Carlos Medicis Morel, David William Provance, Flávio Rocha da Silva

**Affiliations:** 1 Center for Technological Development in Health (CDTS)/National Institute of Science and Technology for Innovation in Neglected Population Diseases (INCT-IDPN), Oswaldo Cruz Foundation (FIOCRUZ), Rio de Janeiro 21040-900, Brazil; carlos.morel@fiocruz.br (C.M.M.); bill.provance@fiocruz.br (D.W.P.J.); flavio.rocha@ioc.fiocruz.br (F.R.d.S.); 2 Technological Center of the Marine Corps (CTecFN), Brazilian Navy, Rio de Janeiro 21010-076, Brazil; andreia.carneiro@marinha.br (A.C.d.S.); marianne.manerat@marinha.br (M.M.M.); 3 Program of Post-Graduation on Science and Biotechnology, Department of Molecular and Cellular Biology, Biology Institute, Federal Fluminense University, Niteroi 22040-036, Brazil; 4 Program of Post-Graduation on Parasitic Biology, Oswaldo Cruz Institute, Oswaldo Cruz Foundation, Rio de Janeiro 21040-900, Brazil

**Keywords:** viral hemorrhagic fevers, diagnostic preparedness, biosafety and biosecurity, military medicine, biodefense surveillance

## Abstract

Viral hemorrhagic fevers (VHFs) are severe infectious diseases caused by RNA viruses of the families Arenaviridae, Filoviridae, Flaviviridae, and Hantaviridae, characterized by high morbidity, significant case fatality rates, and frequent diagnostic uncertainty in early disease stages. For military medical services, timely clinical recognition and laboratory confirmation are essential to guide patient management, prevent nosocomial transmission, and maintain operational continuity, particularly in endemic or resource-limited deployment settings. This review critically examines current diagnostic approaches to VHF-causative agents, emphasizing their use in clinical and field medical settings. The diagnostic process, from exposure through specimen collection, laboratory testing, and result interpretation is analyzed, including the use of molecular, serological, and antigen-based assays. Particular attention is given to deployable diagnostic platforms and their role in bridging the gap between frontline clinical suspicion and definitive laboratory confirmation. Biosafety requirements and infection prevention measures are discussed as integral components of clinical diagnostic workflows, aligned with guidance from the World Health Organization and the Centers for Disease Control and Prevention. Comparative analyses of virus-specific diagnostic timelines and laboratory requirements are presented to support differential diagnosis and clinical decision-making. Emerging technologies, including rapid molecular assays and genomic methods, are evaluated for their potential to improve early diagnosis and patient outcomes. This review highlights the central role of diagnostic readiness in clinical management of the VHFs and provides evidence-based considerations to support military clinicians facing high-risk febrile illnesses in operational environments.

## 1. Introduction

### 1.1. Global Biosecurity Context, Military Relevance, and Threat Landscape

Viral hemorrhagic fevers (VHFs) are a group of severe, often fatal zoonotic diseases caused by enveloped RNA viruses that belong predominantly to the families Filoviridae, Arenaviridae, Nairoviridae, Phenuiviridae, Flaviviridae, and Hantaviridae. Despite substantial virological diversity, these pathogens share convergent clinical and operational characteristics that place them among the highest-priority biological threats to global health security and military medical systems. These include the capacity for rapid human-to-human transmission, high case fatality rates, the absence or limited availability of licensed therapeutics or vaccines for many agents, and stringent biosafety requirements for laboratory handling and diagnosis [1,2,3].

From a military medicine perspective, VHFs occupy a unique position at the intersection of infectious disease epidemiology, biodefense, operational readiness, and international security. Armed forces are disproportionately exposed to VHF risks due to deployment in endemic or outbreak-prone regions, humanitarian and peacekeeping missions, and to disaster response operations, where healthcare infrastructure may be degraded or overwhelmed. Moreover, the deliberate misuse of VHF agents as biological weapons, while historically limited, remains a persistent concern within national and multinational defense planning frameworks [4,5].

The strategic relevance of VHFs is underscored by their consistent inclusion in high-consequence pathogen lists maintained by the World Health Organization (WHO), the U.S. Centers for Disease Control and Prevention (CDC), and military alliances such as NATO. Filoviruses, including Ebola virus and Marburg virus, and arenaviruses such as Lassa virus, are classified as Risk Group 4 agents due to their extreme pathogenicity and lack of widely available countermeasures [2,6]. Several arboviral VHFs, including dengue virus, yellow fever virus, and Crimean–Congo hemorrhagic fever virus, pose additional challenges due to vector-borne transmission, geographic expansion driven by climate change, and the potential for simultaneous civilian and military exposure during outbreaks [7,8,9].

Historically, VHF outbreaks have destabilized healthcare systems, disrupted military operations, and generated significant political and societal consequences. The 2014–2016 West African Ebola virus disease epidemic exemplified these dynamics, resulting in more than 28,000 cases, over 11,000 deaths, and the deployment of military medical assets from multiple nations to support outbreak containment, diagnostics, and logistics [10,11]. Similar operational lessons have emerged from outbreaks of Lassa fever in West Africa, Crimean–Congo hemorrhagic fever in Eastern Europe and Central Asia, and yellow fever in sub-Saharan Africa and South America [12,13,14].

The biological characteristics of VHF agents complicate both clinical recognition and laboratory diagnosis. Early symptoms are typically nonspecific and include fever, malaise, headache, and myalgia, and can closely resemble malaria, bacterial sepsis, or other endemic febrile illnesses. Hemorrhagic manifestations, when present, often occur late in the disease course and are neither universal nor pathognomonic [15]. Consequently, delayed clinical suspicion remains a major contributor to nosocomial transmission, laboratory exposure incidents, and delayed outbreak detection [16].

In military and austere settings, diagnostic challenges are amplified by limited laboratory infrastructure, constraints on specimen transport, and the need to protect medical personnel and maintain operational continuity. The requirement for rapid, accurate, and biosafe diagnostic workflows is therefore not merely a clinical concern but a strategic imperative. Delays or failures in diagnosis can lead to inappropriate patient management, uncontrolled transmission within military units, and mission degradation [17,18].

Biosecurity considerations extend beyond individual patient diagnosis to encompass the entire diagnostic chain, from exposure assessment and specimen collection to laboratory testing, data reporting, and decision-making. VHFs demand a risk-based approach to biosafety that integrates technical containment measures, procedural controls, personnel training, and command-level oversight. International standards, such as the WHO Laboratory Biosafety Manual and the CDC/NIH Biosafety in Microbiological and Biomedical Laboratories (BMBL), provide the foundation for these practices, but their implementation must be adapted to military operational realities [2,6].

The dual-use nature of diagnostic technologies further complicates VHF biosecurity [19]. Molecular platforms capable of detecting high-consequence pathogens can support outbreak response and surveillance but also raise concerns about misuse, data security, and pathogen information hazards. Military medical systems must therefore balance transparency and international collaboration with the need to safeguard sensitive capabilities and information [20].

Advances in diagnostic technologies over the past two decades have significantly transformed the detection and management of VHFs. Real-time reverse transcription polymerase chain reaction (RT-PCR) has become the cornerstone of acute-phase diagnosis, offering high sensitivity and specificity when performed on appropriately collected specimens [21]. Next-generation sequencing has enhanced outbreak investigation, pathogen characterization, and attribution, while rapid diagnostic tests and portable molecular platforms have expanded diagnostic capacity in field settings [22,23,24].

Despite these advances, diagnostic inequities persist, particularly in regions where VHFs are endemic. Limited access to high-containment laboratories, shortages of trained personnel, and fragile supply chains continue to impede timely diagnosis and outbreak control. For military forces operating in such environments, reliance on host-nation diagnostic capacity may be insufficient, necessitating deployable laboratory assets and multinational cooperation [25].

Military doctrine increasingly recognizes infectious disease threats as integral components of the operational environment. Modern concepts of force health protection emphasize early detection, situational awareness, and rapid response to biological hazards, including naturally occurring outbreaks and deliberate biological incidents. VHFs, due to their severity and complexity, serve as critical test cases for integrating medical intelligence, diagnostics, and command decision-making [5,26].

This review aims to provide a comprehensive, strategically oriented analysis of the biosecurity and diagnostic dimensions of viral hemorrhagic fevers, with a particular focus on military medical relevance. It synthesizes current international guidance, peer-reviewed evidence, and operational experience to outline best practices for diagnosis, laboratory biosafety, and deployment of diagnostic capabilities. By integrating virological, clinical, and biosecurity perspectives, the review seeks to support informed decision-making by military medical planners, laboratory leaders, and policymakers.

The subsequent sections will examine virus-specific diagnostic strategies, the comparative performance of diagnostic platforms, biosafety-level requirements and laboratory containment, and the integration of centralized and deployable diagnostic systems. An overarching diagnostic and biosecurity workflow is presented to contextualize these elements within a coherent operational framework applicable to both civilian outbreak response and military operations.

### 1.2. Virus-Specific Diagnostic Strategies and Operational Platforms

Accurate diagnosis of viral hemorrhagic fevers depends on a precise understanding of the causative virus, the stage of infection at which clinical suspicion arises, and the biosafety constraints under which diagnostic testing is performed. Unlike many common infectious diseases, VHFs demand a diagnostic approach that integrates molecular sensitivity with stringent containment requirements, particularly in military and field environments where laboratory infrastructure may be limited. Virus-specific diagnostic strategies therefore represent a cornerstone of both clinical management and biosecurity risk mitigation.

#### 1.2.1. Filoviridae: Ebola Virus and Marburg Virus

Filoviruses remain the archetypal VHF pathogens due to their high lethality, epidemic potential, and historical role in shaping global outbreak response doctrine. Diagnostic confirmation of Ebola virus disease (EVD) and Marburg virus disease relies primarily on real-time reverse transcription polymerase chain reaction (RT-PCR) targeting conserved genomic regions such as nucleoprotein (NP), glycoprotein (GP), or RNA-dependent RNA polymerase (L) genes. RT-PCR assays demonstrate high analytical sensitivity during the acute viremic phase, often detectable within the first three days of symptom onset [21,27,28].

Antigen detection assays, including lateral flow immunoassays targeting viral nucleoproteins, have been deployed in outbreak settings to support rapid triage and isolation decisions. While these assays offer operational advantages in speed and minimal equipment requirements, their reduced sensitivity compared with RT-PCR necessitates confirmatory molecular testing, particularly for low-viral-load samples or early infection [23].

Serological diagnostics play a limited role in acute filovirus infection due to delayed antibody responses and high mortality. IgM and IgG enzyme-linked immunosorbent assays (ELISA) are primarily used for retrospective diagnosis, survivor follow-up, and seroepidemiological studies [29]. Virus isolation and culture are restricted to biosafety level 4 (BSL-4) laboratories and are not routinely performed for diagnostic purposes because of the extreme risk posed by handling live virus [2].

From a military medical standpoint, filovirus diagnostics underscore the need for deployable molecular platforms that can operate under high-containment field conditions. Mobile laboratories equipped with negative-pressure gloveboxes and portable RT-PCR systems have proven effective in reducing diagnostic turnaround times during outbreaks and supporting force health protection in deployed settings [30].

#### 1.2.2. Arenaviridae: Lassa Virus and South American Arenaviruses

Arenaviruses, particularly Lassa virus, pose a persistent threat in West Africa and cause significant morbidity among civilian populations and deployed personnel. Diagnostic confirmation relies on RT-PCR detection of viral RNA in blood, serum, or plasma during the acute phase of illness. Lassa virus exhibits genetic diversity across lineages, necessitating careful assay design to ensure broad coverage and minimize false-negative results [31].

Antigen detection assays for Lassa virus have been developed but are not widely available or standardized. Serological assays, including IgM and IgG ELISA, are useful for diagnosing infection in later stages and for surveillance. However, cross-reactivity with other arenaviruses and delayed seroconversion limit their utility as standalone diagnostic tools [32].

South American arenaviruses, such as Junín, Machupo, and Guanarito viruses, are geographically restricted but pose a severe disease risk with high case fatality rates. Diagnostic principles parallel those for Lassa virus, with molecular detection as the primary modality and serology as a complementary approach. As with filoviruses, virus isolation is confined to BSL-4 laboratories and is rarely indicated outside research or reference settings [33].

#### 1.2.3. Nairoviridae (Crimean–Congo Hemorrhagic Fever Virus) and Phenuiviridae (Order Bunyavirales)

Tick-borne viral hemorrhagic fevers represent a significant and operationally relevant group of zoonotic high-consequence infections, with Crimean–Congo hemorrhagic fever virus (CCHFV) as the most prominent and widely distributed agent across Africa, the Middle East, Asia, and parts of Europe. Transmitted primarily by *Hyalomma* spp. ticks, CCHFV poses a persistent threat to both civilian and military populations, particularly in endemic regions. The emergence of additional tick-borne phleboviruses (Plenuiviridae), such as Severe Fever with Thrombocytopenia Syndrome virus (SFTSV) in Asia and Heartland virus in North America, further expands the geographic and epidemiological importance of this group. Clinically, these infections are characterized by nonspecific early manifestations, including fever, thrombocytopenia, and leukopenia, which hinder timely recognition and may delay appropriate infection control measures. The combined risk of vector-borne transmission and potential human-to-human spread, especially through exposure to infected blood or bodily fluids, complicates outbreak management and biosecurity [14].

CCHFV, in particular, presents distinct diagnostic and operational challenges due to its broad distribution and capacity to cause nosocomial outbreaks. RT-PCR remains the gold standard for early detection during the acute phase, typically targeting conserved regions such as the small (S) genomic segment, while serological assays (e.g., IgM ELISA) become informative after the first week of illness, especially in settings with limited molecular capacity [34]. Non-propagative diagnostic procedures can be performed under biosafety level 3 conditions with appropriate precautions. In endemic and high-risk settings, particularly in military operations, integrating tick-borne VHF diagnostics into routine febrile-illness algorithms, combined with surveillance and vector-control strategies, is essential to ensure rapid case identification, reduce transmission risk, and maintain operational readiness.

#### 1.2.4. Flaviviridae: Dengue Virus and Yellow Fever Virus

Flavivirus VHFs, including dengue virus (DENV) and yellow fever virus (YFV), differ from filoviruses and arenaviruses in their transmission dynamics [35] and diagnostic profiles [36]. Dengue virus infection is characterized by high viremia early in the illness, allowing RT-PCR detection within the first 5 days after symptom onset. Non-structural protein 1 (NS1) antigen detection assays are a rapid, widely used diagnostic option in both civilian and military contexts [37].

Serological assays for dengue, while essential for diagnosis after the acute phase, are complicated by extensive cross-reactivity among flaviviruses [38,39] and coronaviruses [40], particularly in individuals with prior vaccination or infection. This limitation has significant implications for military personnel deployed to regions where multiple flaviviruses co-circulate [41].

Diagnosis of YFV similarly relies on RT-PCR during early infection and on serology thereafter. Vaccination history must be carefully considered when interpreting serological results, as vaccine-induced antibodies can confound diagnostic interpretation [42].

#### 1.2.5. Hantaviridae and Phenuiviridae

Hantaviruses and phleboviruses, including Rift Valley fever virus, present additional diagnostic considerations. RT-PCR and serology are both employed, with the choice of modality guided by disease phase and specimen availability. Rift Valley fever virus, due to its zoonotic and epizootic nature, is of particular relevance to military operations involving livestock or vector exposure [43].

Table 1 summarizes virus-specific diagnostic approaches, specimen types, biosafety requirements, and primary use cases. This comparative framework supports informed diagnostic selection and risk assessment across diverse operational scenarios.

## 2. Centralized Versus Deployable Diagnostic Platforms

The operational context strongly influences the selection of a diagnostic platform. Centralized reference laboratories offer comprehensive diagnostic capabilities, including confirmatory testing, viral sequencing, and quality assurance. However, they are limited by specimen transport times and dependency on secure logistics [44].

Deployable diagnostic platforms, including mobile laboratories and point-of-care molecular systems, provide rapid preliminary diagnosis in outbreak or combat settings. While these systems may lack the full analytical breadth of centralized laboratories, they play a critical role in early detection, isolation, and force protection [45]. Table 2 compares centralized and deployable diagnostic platforms in terms of performance, biosafety, and operational suitability.

## 3. Biosafety, Containment Doctrine, and Biosecurity Risk Management

The diagnosis of VHF is inseparable from biosafety and biosecurity considerations. Unlike routine clinical microbiology, VHF diagnostics must balance analytical performance with strict containment measures designed to protect laboratory personnel, healthcare workers, and surrounding communities. In military medical systems, these requirements are further shaped by operational constraints, expeditionary environments, and the potential use of VHFs as biological threats.

### 3.1. Biosafety Levels and Containment Principles

International biosafety frameworks, including those established by the WHO, the United States CDC, and military medical doctrine, classify laboratory activities according to biosafety levels (BSL) based on pathogen transmissibility, disease severity, and the availability of effective countermeasures. VHFs span multiple biosafety categories, with filoviruses and certain arenaviruses requiring the highest level of containment. Work involving Ebola virus, Marburg virus, and Lassa virus requires biosafety level 4 (BSL-4) facilities, which incorporate maximum containment measures such as positive-pressure suits, dedicated air-supply systems, controlled access, and chemical decontamination processes [2]. While essential for viral culture, pathogenesis studies, and countermeasure development, BSL-4 laboratories are limited in number and are not suited for routine diagnostic throughput.

In contrast, non-propagative diagnostic procedures such as molecular testing on properly inactivated specimens can be safely conducted under BSL-3 conditions when validated inactivation protocols are applied [46]. This approach has significantly expanded access to molecular diagnostics during outbreaks, allowing broader laboratory participation beyond specialized high-containment centers. Virus isolation by cell culture, although a definitive diagnostic method, is rarely used in routine practice due to its requirement for BSL-4 containment, prolonged turnaround time, and lower sensitivity in early infection. Consequently, its use is largely restricted to specialized reference laboratories for research and outbreak investigations. In clinical and operational settings, including military environments, rapid and safe diagnostic strategies rely primarily on RT-PCR and antigen-based assays, which offer a more practical balance between performance, biosafety, and timeliness.

### 3.2. Biosafety in Molecular Diagnostics

Molecular diagnostics represent the backbone of modern VHF detection, but they introduce specific biosafety challenges related to specimen handling, aerosol generation, and waste management. Pre-analytical steps, including blood collection, centrifugation, and nucleic acid extraction, pose the highest risk of exposure. Accordingly, WHO and CDC guidelines emphasize the use of sealed centrifuge rotors, class II or III biological safety cabinets, and chemical or thermal inactivation prior to downstream processing [1,47].

For military laboratories, especially those deployed forward, adherence to these principles requires careful platform selection and personnel training. Compact molecular systems with closed-cartridge designs reduce operator exposure and simplify biosafety compliance, albeit sometimes at the expense of assay flexibility or throughput [48].

### 3.3. Occupational Risk and Laboratory-Acquired Infections

A historical analysis of laboratory-acquired infections underscores the dangers of VHF diagnostics when biosafety measures are inadequate. Crimean–Congo hemorrhagic fever virus has been associated with multiple laboratory and healthcare worker infections, often linked to needlestick injuries or unrecognized exposure to infectious blood products [49,50].

Filoviruses, while less commonly associated with laboratory-acquired infections, present catastrophic consequences in the event of containment failure. As a result, military medical doctrine emphasizes redundancy in engineering controls, procedural safeguards, and medical surveillance of laboratory personnel handling high-risk specimens [51].

### 3.4. Biosecurity and Dual-Use Considerations

Beyond biosafety, VHF diagnostics intersect with broader biosecurity concerns, particularly the potential misuse of pathogens or diagnostic knowledge for hostile purposes. VHFs are recognized under international frameworks, including the Biological Weapons Convention, as agents of concern due to their lethality and psychological impact.

Diagnostic laboratories, especially those operating in conflict zones or politically unstable regions, must therefore implement robust biosecurity measures. These include controlled access to specimens and data, chain-of-custody documentation, and coordination with military intelligence and public health authorities when unusual disease patterns are detected.

### 3.5. Military-Specific Containment Doctrine

Military medical systems adopt a layered approach to biosafety and biosecurity, integrating civilian public health standards with force protection requirements. This approach recognizes that deployed forces may encounter VHFs in environments lacking established healthcare infrastructure, necessitating self-sufficient diagnostic and containment capabilities.

Mobile high-containment laboratories, often deployed under military or joint civilian–military command, exemplify this doctrine. These units combine modular laboratory design, negative-pressure workspaces, and on-site waste decontamination to enable safe diagnostics in austere settings [52]. While not equivalent to fixed BSL-4 facilities, such platforms provide a critical bridge between frontline clinical care and centralized reference laboratories. Table 3 summarizes biosafety levels, containment requirements, and laboratory capabilities for major VHF agents, harmonized with WHO, CDC, and military doctrine.

### 3.6. Waste Management and Environmental Decontamination

Effective waste management is a critical but often underemphasized component of VHF diagnostics. Liquid waste, sharps, and disposable PPE must be decontaminated with validated chemical disinfectants or by autoclaving prior to disposal. Environmental persistence of certain VHF agents necessitates rigorous surface decontamination protocols, particularly in field laboratories where infrastructure may be limited [53,54].

Military guidelines further emphasize the need for contingency planning in the event of laboratory compromise, including evacuation procedures, environmental remediation, and, where available, post-exposure prophylaxis.

### 3.7. Ethical and Legal Dimensions

The handling of VHF specimens raises ethical and legal considerations related to informed consent, data sharing, and international cooperation. During multinational military operations or humanitarian deployments, diagnostic activities must comply with host-nation regulations and international standards. Transparent communication between military medical units, civilian health authorities, and international organizations is essential to maintain trust and ensure effective outbreak response [55].

### 3.8. Diagnostic Approaches Across Viral Hemorrhagic Fevers

The diagnosis of VHFs relies on a combination of molecular, antigen-based, and serological assays, with the choice of method determined by the causative virus, stage of infection, and available laboratory capacity (Table 4). RT-PCR remains the gold standard for most VHFs, particularly during the acute phase of illness, due to its high sensitivity and specificity for detecting viral RNA. This approach is central to the diagnosis of filoviruses (Ebola and Marburg), arenaviruses (Lassa), and nairoviruses (Crimean–Congo hemorrhagic fever), where early viremia is prominent [21].

Antigen detection assays, including RDTs and ELISA, provide valuable tools for early screening, especially in outbreak or field settings. For example, NS1 antigen detection in dengue and nucleoprotein-based assays for Ebola virus enable rapid triage and case identification. However, these assays generally exhibit lower sensitivity than RT-PCR and may yield false-negative results, particularly at low viral loads [27,28].

Serological assays detecting IgM and IgG antibodies play a complementary role, becoming more informative during the later stages of infection or in retrospective diagnosis. These methods are widely used for dengue, hantavirus, and yellow fever infections, although cross-reactivity among related viruses, particularly within the Flaviviridae family, can complicate interpretation. Confirmatory assays, such as plaque reduction neutralization tests (PRNT), are often required in such cases.

Overall, optimal diagnostic strategies for VHFs require a staged, integrated approach that combines rapid screening methods with confirmatory molecular testing while accounting for biosafety constraints and operational context.

### 3.9. Diagnostic Timing and Implications

The diagnostic accuracy of VHFs is strongly influenced by the temporal relationship between infection, symptom onset, and specimen collection (Table 4). Incubation periods vary widely across VHF agents, ranging from 1 to 9 days for Crimean–Congo hemorrhagic fever to up to three weeks for filoviruses and arenaviruses. During this asymptomatic phase, laboratory detection is generally not feasible, emphasizing the importance of exposure assessment and clinical vigilance.

Following symptom onset, nucleic acid detection by RT-PCR provides the earliest and most reliable confirmation for most VHFs, typically within the first week of illness when viremia is highest [23]. However, this window is relatively short for some pathogens, such as dengue and yellow fever viruses, where viral RNA may decline rapidly after day 5 [56,57]. In contrast, filoviruses and Lassa virus often maintain detectable viremia for longer periods, extending the utility of molecular diagnostics.

Serological responses, particularly IgM antibodies, generally emerge between days 4 and 7 after symptom onset and serve as an important complementary diagnostic tool when molecular detection is no longer available. IgG antibodies appear later and are primarily useful for retrospective diagnosis and epidemiological studies. However, cross-reactivity, especially among flaviviruses, remains a significant limitation [39,40].

These temporal dynamics highlight the necessity of a staged diagnostic approach that integrates molecular and serological methods according to disease phase. In operational and military settings, failure to account for these detection windows may lead to false negatives and delayed outbreak detection, underscoring the importance of repeat testing and combined diagnostic strategies.

## 4. Integration into Outbreak Response and Force Protection

Ultimately, biosafety and biosecurity measures are not ends in themselves but integral components of a broader outbreak response strategy. Timely diagnosis, safe specimen handling, and secure data management enable rapid clinical decision-making, targeted infection control, and effective communication with command structures.

For military forces, integrating diagnostic biosafety into operational planning enhances resilience against both naturally occurring outbreaks and deliberate biological threats. The lessons learned from recent VHF outbreaks highlight the necessity of sustained investment in training, infrastructure, and international collaboration [58].

### 4.1. Diagnostic Timelines, Clinical Algorithms, and Operational Workflows

Timely diagnosis of VHFs is a decisive factor in patient outcomes, outbreak containment, and force protection. Unlike many infectious diseases, VHFs present a narrow window in which early clinical suspicion must be translated into laboratory confirmation under heightened biosafety conditions. Diagnostic delays not only increase mortality risk but also amplify the potential for secondary transmission among healthcare workers and military personnel. Consequently, modern VHF diagnostic doctrine emphasizes time-structured workflows that integrate exposure history, clinical evolution, specimen selection, and laboratory capacity.

### 4.2. Temporal Dynamics of VHF Infection

The incubation periods of VHFs vary by virus family but typically range from 2 to 21 days. During this phase, individuals are asymptomatic, and diagnostic testing is generally uninformative. Once symptoms emerge, viral kinetics diverge significantly across pathogens, shaping the optimal diagnostic approach.

Filoviruses and arenaviruses are characterized by rapidly increasing viremia shortly after symptom onset, rendering molecular diagnostics highly sensitive during the first week of illness. In contrast, flaviviral VHFs such as dengue may exhibit an earlier but shorter window of detectable viremia, followed by a rapid transition to antibody-mediated diagnosis. These temporal differences necessitate pathogen-specific diagnostic algorithms that are responsive to both disease stage and operational context [59].

### 4.3. Clinical Suspicion and Case Definition

Initial diagnostic pathways begin with clinical suspicion based on non-specific symptoms—fever, malaise, headache, myalgia—that overlap with numerous endemic diseases. In military deployments, differential diagnosis must also consider malaria, typhoid fever, leptospirosis, and other febrile illnesses common in austere environments.

Standardized case definitions, such as those issued by the WHO and CDC, provide structured criteria for suspect, probable, and confirmed VHF cases. While essential for surveillance and reporting, these definitions are intentionally conservative and may delay laboratory testing if applied rigidly. Military medical doctrine therefore advocates a lower threshold for initiating diagnostic testing when the risk of exposure is credible, particularly after contact with wildlife, ticks, livestock, or known outbreak zones [1,60].

### 4.4. Specimen Selection and Handling over Time

Specimen type and timing are critical determinants of diagnostic yield. Whole blood, plasma, or serum collected during the acute febrile phase provides the highest sensitivity for RT-PCR-based assays. Improper timing or specimen choice can lead to false negatives, particularly early in the disease, when viral loads may be near the detection threshold.

As infection progresses, serological assays gain diagnostic value, with IgM antibodies typically detectable from days 5 to 7 onward. However, reliance on serology alone is discouraged in high-consequence settings due to delayed seroconversion and cross-reactivity. Sequential testing strategies combining molecular and serological assays are therefore recommended to maximize diagnostic confidence [61].

### 4.5. Diagnostic Turnaround Time and Decision-Making

In outbreak and military settings, diagnostic turnaround time directly influences isolation decisions, resource allocation, and command-level risk assessments. Centralized laboratories offer high analytical performance but may require days for specimen transport and result reporting. Deployable diagnostic platforms reduce this delay, enabling same-day or next-day results that support rapid clinical and operational decisions.

**Figure 1:**
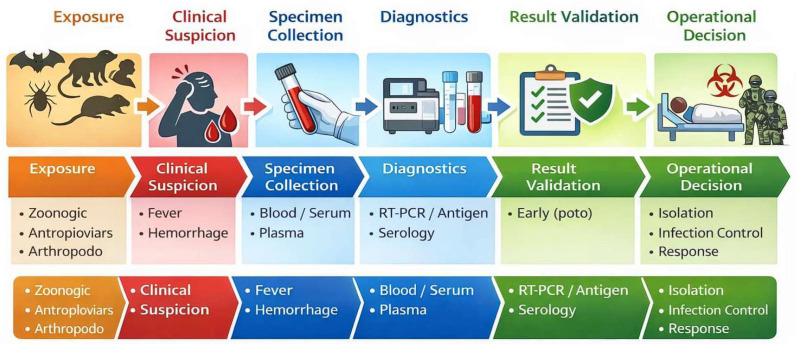
Diagnostic and biosecurity workflow for viral hemorrhagic fevers. The workflow begins with an exposure assessment that incorporates epidemiological intelligence, travel history, and operational context. This step informs early risk stratification and the activation of precautionary measures, even before symptom onset. Following clinical presentation, immediate infection prevention and control measures are implemented, including patient isolation and use of appropriate personal protective equipment. Specimen collection is performed using standardized protocols designed to minimize exposure risk, with immediate inactivation where feasible. Laboratory processing proceeds through validated containment pathways, ranging from BSL-2 to BSL-4, depending on pathogen and procedure. Finally, the interpretation and communication of results feed into clinical management, contact tracing, and command-level situational awareness.

However, rapid diagnostics introduce trade-offs between speed, sensitivity, and biosafety. Field-deployed molecular platforms must be rigorously validated and integrated into broader laboratory networks to ensure confirmatory testing and epidemiological oversight [62]. Figure 1 illustrates the integrated diagnostic and biosecurity workflow from exposure to result, emphasizing critical control points where clinical, laboratory, and biosafety decisions intersect.

### 4.6. Clinical Algorithms for Military Settings

Military diagnostic algorithms prioritize simplicity, robustness, and adaptability. Unlike civilian tertiary care centers, deployed medical units must operate with limited personnel, equipment, and time. Algorithms therefore emphasize early molecular testing for high-risk cases, coupled with conservative infection control measures until VHF is excluded.

Decision trees commonly integrate exposure risk, symptom severity, and diagnostic availability to guide escalation from field testing to centralized confirmation. Importantly, negative initial results do not automatically rule out VHF, particularly if testing occurs early in the disease course. Repeat testing at 24–48 h intervals is recommended when clinical suspicion remains high [63].

### 4.7. Communication, Reporting, and Learning from Outbreak Responses

Effective communication of diagnostic results is essential for outbreak control and military command decision-making. Laboratories must establish secure, redundant reporting channels to ensure the timely dissemination of results to clinicians, infection control teams, and operational leadership. Delays or ambiguity in the communication of results can undermine trust and impede coordinated response efforts.

Recent VHF outbreaks have highlighted recurring diagnostic challenges, including delayed testing, insufficient biosafety training, and fragmented laboratory networks. Conversely, successful responses demonstrate the value of pre-established diagnostic workflows, integration with mobile laboratories, and continuous training for medical personnel [64].

For military medical systems, these lessons reinforce the need for preparedness throughout the diagnostic timeline, from exposure assessment to definitive confirmation. Diagnostic excellence in VHFs is not merely a technical achievement but a strategic capability that underpins biosecurity and force protection.

## 5. Strategic Preparedness, Future Diagnostic Directions, and Military Biosecurity Integration

The sustained threat posed by VHFs necessitates a forward-looking diagnostic and biosecurity strategy that transcends reactive outbreak response. For military medical systems, preparedness for VHFs represents both a public health imperative and a core element of biological defense. Advances in diagnostics, coupled with evolving operational environments and emerging technologies, require continuous adaptation of doctrine, infrastructure, and training.

### 5.1. Strategic Role of Diagnostics in Biosecurity

Diagnostics occupy a central position in the biosecurity architecture surrounding VHFs. Early detection enables timely clinical intervention, limits secondary transmission, and supports strategic decision-making at both tactical and operational levels. In military contexts, diagnostic confirmation of a VHF case may trigger force protection measures, mission reconfiguration, or coordination with civilian health authorities. Beyond individual patient care, diagnostics contribute to surveillance and situational awareness; when integrated with syndromic surveillance systems, laboratory confirmation provides early warning of outbreaks that may threaten deployed forces or destabilize regions of strategic interest [65].

Despite these critical functions, important limitations affect the performance and interpretation of available diagnostic tools. Rapid diagnostic tests, particularly antigen-based lateral flow assays, offer significant operational advantages, including speed, ease of use, and minimal infrastructure requirements. However, these benefits are offset by reduced analytical sensitivity compared with molecular assays. While specificity is generally high, making positive results useful for rapid triage and isolation, false-negative results remain a major concern, especially during early infection or in cases with low viral load. This limitation is particularly relevant in high-risk settings, where premature exclusion of VHF based on a single negative RDT may compromise infection control measures.

RRT-PCR remains the reference standard for VHF diagnosis due to its superior sensitivity and specificity. Nevertheless, RT-PCR is not infallible. False-negative results may occur due to improper specimen collection, testing outside the optimal detection window, degradation of viral RNA, or technical and logistical constraints, which are amplified in field and resource-limited environments. Additionally, the requirement for specialized equipment, trained personnel, and biosafety infrastructure limits its immediate availability in austere or rapidly evolving operational settings.

The trade-off between sensitivity and specificity across diagnostic platforms necessitates a cautious, integrated approach to interpreting results. In practice, this involves combining rapid tests for initial screening with confirmatory RT-PCR, alongside repeat testing in cases of persistent clinical suspicion. From a biosecurity perspective, the consequences of false-negative results extend beyond individual patients, potentially enabling undetected transmission chains and undermining operational readiness. Therefore, diagnostic strategies for VHFs must prioritize sensitivity within a layered testing framework, ensuring that clinical judgment, epidemiological context, and laboratory data are jointly considered in decision-making processes.

### 5.2. Preparedness Frameworks and Doctrine Alignment

Preparedness for VHF diagnostics is guided by a convergence of civilian public health frameworks and military medical doctrine. WHO and CDC guidelines establish foundational principles for laboratory readiness, while military-specific doctrines emphasize scalability, redundancy, and interoperability.

Key components of preparedness include pre-positioning of diagnostic reagents, maintaining deployable laboratory units, and establishing referral pathways to regional or international reference laboratories. Exercises and simulations play a critical role in validating these systems and in exposing gaps in logistics, communication, and biosafety compliance before real-world crises occur [66].

### 5.3. Training and Workforce Development

Human expertise remains a limiting factor in VHF diagnostics, particularly in high-containment laboratory operations. Military medical personnel must be proficient not only in technical diagnostic procedures but also in biosafety practices, risk assessment, and emergency response.

Training programs increasingly emphasize cross-disciplinary competencies, integrating laboratory science with clinical medicine, epidemiology, and biosecurity. Simulation-based training, including mock specimen handling and containment breach scenarios, enhances preparedness and reduces the risk of laboratory-acquired infections [67].

### 5.4. Emerging Diagnostic Technologies

Technological innovation is reshaping the diagnostic landscape for VHFs. Next-generation sequencing (NGS) platforms enable comprehensive pathogen identification and genomic surveillance, supporting outbreak tracing and detection of novel or engineered variants. While currently confined largely to centralized laboratories, miniaturized sequencing platforms are increasingly being evaluated for field deployment [68,69].

Clustered Regularly Interspaced Short Palindromic Repeats (CRISPR)-based diagnostics represent another promising avenue, offering rapid, highly specific detection with minimal equipment requirements. Early studies demonstrate potential applicability to filoviruses and other high-consequence pathogens, although validation under field conditions remains ongoing [70].

Digital health technologies, including artificial intelligence-driven [71,72,73] decision-support systems, further enhance diagnostic workflows by integrating clinical data, exposure risk, and laboratory results into coherent risk assessments. Such systems may prove particularly valuable in resource-limited or high-tempo military operations.

Metagenomic and nanopore-based sequencing are also alternative approaches that have become increasingly relevant for the diagnosis and surveillance of VHFs, particularly for high-consequence pathogens such as the Ebola virus, Lassa virus, and CCHFV. During the West African Ebola outbreak, real-time genomic sequencing enabled rapid characterization of viral transmission chains and supported outbreak containment efforts, establishing a paradigm for integrating sequencing into field response [74,75]. More recently, portable nanopore platforms have facilitated near-real-time, on-site sequencing in resource-limited and high-risk environments, allowing simultaneous pathogen detection and genomic surveillance without reliance on centralized laboratories [76,77]. For Lassa fever and CCHFV, sequencing approaches have been instrumental in identifying genetic diversity, tracking viral evolution, and improving molecular assay design, particularly in regions with high strain variability [78,79,80].

Despite these advances, challenges remain, including the need for standardized protocols, robust bioinformatics pipelines, and biosafety-compliant workflows for handling high-risk specimens. Sensitivity may be affected by low viral loads and high background host nucleic acid levels, necessitating optimized sample preparation strategies, such as host depletion. Nevertheless, the integration of field-deployable sequencing into VHF diagnostics represents a significant advancement in outbreak response, enhancing early detection, supporting epidemiological investigations, and strengthening biosecurity and military readiness in endemic and emerging threat settings [80,81,82].

Wastewater and environmental surveillance have become essential tools for monitoring high-risk pathogens, enabling early detection, genomic tracking, and population-level assessment of infectious diseases. Recent studies demonstrate their utility in tracking SARS-CoV-2 evolution, detecting emerging viruses such as Mpox, and monitoring respiratory pathogens, including Influenza A, highlighting their role as complementary systems to clinical diagnostics and genomic surveillance in modern biosecurity frameworks [83,84,85,86,87,88,89].

### 5.5. Integration with Global Health Security

Military medical capabilities play a unique role in global health security [90], often bridging diagnostic capacity gaps during international outbreaks. Deployable laboratories and trained personnel have supported civilian health systems during Ebola outbreaks in West Africa and elsewhere, illustrating the dual-use nature of military diagnostic assets.

This integration requires careful coordination to ensure alignment with international norms, respect for sovereignty, and ethical use of military resources in public health emergencies. Transparent collaboration enhances trust and strengthens collective resilience against VHF threats [90].

### 5.6. Biosecurity and Counterproliferation Considerations

The diagnostic domain intersects with counterproliferation efforts aimed at preventing the misuse of biological agents. Surveillance data derived from diagnostic testing may provide early indicators of deliberate release or laboratory accidents. Military medical intelligence units therefore increasingly collaborate with diagnostic laboratories to contextualize unusual epidemiological patterns [91].

At the same time, dissemination of diagnostic methodologies must be balanced against the risk of dual-use knowledge. Responsible publication practices and controlled access to sensitive protocols form part of a broader biosecurity strategy that safeguards both scientific progress and national security.

### 5.7. Ethical Challenges

Preparedness for VHF diagnostics extends beyond technical considerations to encompass ethical and legal dimensions. Informed consent, data privacy, and equitable access to diagnostics are critical issues, particularly during multinational military operations or humanitarian interventions.

Military medical systems must navigate complex legal frameworks governing specimen transport, data sharing, and international cooperation. Adherence to ethical standards not only protects individual rights but also reinforces legitimacy and trust in military-led health interventions [92].

The evolving threat landscape for VHFs includes climate-driven expansion of vector ranges, increased human–animal interface, and the potential emergence of novel pathogens. These trends underscore the necessity for adaptable diagnostic platforms and flexible biosafety frameworks. Investment in research and development, coupled with sustained international collaboration, will be essential to address these challenges. For military medical systems, maintaining diagnostic readiness for VHFs is not a static goal but an ongoing process that must evolve in step with scientific and geopolitical realities [83,93,94].

### 5.8. Strategy for Biological Defense

Viral hemorrhagic fevers and convergent threats such as the Nipah virus must be recognized as high-consequence biological risks that can simultaneously affect public health, military operational capability, and national and international stability [95,96]. Their high case fatality rates, potential for dissemination, early diagnostic uncertainty, and stringent biosafety requirements justify their prioritization in biological defense frameworks [97], regardless of their specific clinical classification [98].

In defense contexts, safe and timely diagnostic capability constitutes a critical element of operational readiness. Rapid and reliable laboratory confirmation informs strategic decisions related to force protection, asset deployment, continuity of operations, and crisis management. The absence or fragility of such capability undermines evidence-based decision-making, increases the risk of intra-organizational transmission, and may result in destabilizing sanitary, economic, and geopolitical effects [99,100].

The integration of molecular diagnostics, particularly RT-PCR, into deployable medical structures and command-and-control systems should be treated as a strategic priority. A layered diagnostic architecture combining advanced forward-deployed capabilities with centralized reference laboratories ensures operational resilience, redundancy, and strategic oversight. This model applies to both classical VHFs [101] and high-consequence emerging viruses, such as NiV [102,103] (Table 5).

Biosafety and biosecurity constitute inseparable pillars of biological defense. Doctrines and policies should emphasize multilayered containment, protocol standardization, continuous training, and an organizational culture of safety to reduce the risks of occupational exposure, containment failures, and the misuse of biological agents. In addition, diagnostic information must be treated as a strategic asset, requiring robust governance, data protection, and close coordination among defense, health, intelligence, and science and technology sectors (Table 5).

Finally, the incorporation of emerging technologies such as genomic sequencing, rapid diagnostic platforms, CRISPR-based assays, and artificial intelligence [71,72,73] has enabled decision-support systems that enhance early warning and anticipatory response capabilities. Readiness for VHFs and convergent threats such as the NiV directly reflects the level of national biological defense maturity, in which robust diagnostic capabilities, firmly anchored in biosafety and biosecurity, function as decisive force multipliers for force protection, institutional resilience, and global security.

**Table 5:** Comparison between Nipah virus and viral hemorrhagic fevers from a biosafety and preparedness perspective.

Domain	Nipah Virus (NiV)	Viral Hemorrhagic Fevers (VHFs)
Virological family	*Paramyxoviridae* [103,104]	*Filoviridae*, *Arenaviridae*, *Nairoviridae*, *Flaviviridae* [105]
Natural reservoirs	Fruit bats (*Pteropus* spp.) [106]	Rodents, bats, ticks (virus-dependent) [3]
Human-to-human transmission	Documented, mainly healthcare/household settings [105]	Common, especially in healthcare and funerals [106,107]
Case fatality rate	High, often >40% [104,105]	High, virus-dependent [107,108]
Initial clinical features	Nonspecific; respiratory or neurological signs [104]	Nonspecific; may progress to hemorrhage [106,109]
Diagnostic methods	RT-PCR; serology (late phase) [32,105]	RT-PCR; serology; virus isolation [32,45,107]
Biosafety requirements	BSL-3 (diagnostics); BSL-4 (isolation) [24]	BSL-3 or BSL-4, depending on agent [24,110]
Medical countermeasures	Mostly experimental [104]	Available for some VHFs (e.g., Ebola) [110]
Strategic relevance	Emerging high-consequence pathogen [105]	Established high-consequence pathogens [106,109]

## 6. Conclusions

Viral hemorrhagic fevers constitute a persistent biological threat to military forces, civilian populations, and international security. Their high mortality rates, epidemic potential, diagnostic ambiguity, and demanding biosafety requirements position VHFs as pathogens of strategic concern within biodefense and military medical planning. This review examines current diagnostic approaches and biosecurity frameworks for VHF agents, emphasizing their relevance to military operational environments.

Within defense contexts, VHF diagnosis is not merely a clinical task but a critical operational capability. Rapid and reliable laboratory confirmation directly influences force health protection, operational tempo, and command decision-making. Diagnostic failure or delay may degrade mission effectiveness, facilitate intra-unit transmission, and contribute to broader regional destabilization.

Molecular diagnostics, particularly RT-PCR, remain the primary tools for VHF detection across virus families. Their operational value depends on integration into deployable medical assets, time-critical decision pathways, and validated biosafety workflows. Forward-deployable diagnostic platforms enhance situational awareness in austere and contested environments, while centralized reference laboratories provide confirmatory capacity and strategic oversight.

Biosafety and biosecurity are inseparable from diagnostic operations involving high-consequence pathogens. Military medical doctrine emphasizes layered containment, redundancy, and sustained training to mitigate the risk of laboratory-associated exposure or loss of containment. Beyond laboratory settings, diagnostic information supports surveillance, threat attribution, and counterproliferation efforts, requiring secure data handling and coordination with defense, intelligence, and public health authorities.

As environmental change, population mobility, and technological diffusion alter the threat landscape, emerging diagnostic technologies—including genomic sequencing, CRISPR-based assays, and AI-enabled decision support—offer potential advantages for early warning and response. Ultimately, preparedness for VHFs reflects overall biological defense readiness, in which robust diagnostic capability anchored in biosafety and biosecurity serves as a decisive force multiplier for force protection and global stability.

## Figures and Tables

**Table 1:** Virus-by-virus diagnostic comparison for viral hemorrhagic fevers.

Virus Family	Representative Viruses	Primary Diagnostic Method	Specimen	Biosafety Level (Non-Propagative)	Notes
Filoviridae	Ebola, Marburg	RT-PCR	Blood, plasma	BSL-3 (inactivated)	Gold standard: antigen tests for triage
Arenaviridae	Lassa, Junín	RT-PCR, ELISA	Blood, serum	BSL-3	Genetic diversity affects assay design
Nairoviridae	CCHFV	RT-PCR, IgM ELISA	Blood	BSL-3	High nosocomial transmission risk
Flaviviridae	Dengue, Yellow fever	RT-PCR, NS1, ELISA	Serum	BSL-2/3	Cross-reactivity common
Hantaviridae	Hantaan	RT-PCR, ELISA	Serum	BSL-2/3	Often diagnosed serologically
Phenuiviridae	Rift Valley fever	RT-PCR, ELISA	Blood	BSL-3	Zoonotic and vector-borne

**Table 2:** Diagnostic platforms for VHFs: centralized versus deployable.

Parameter	Centralized Laboratories	Deployable Platforms
Diagnostic scope	Comprehensive	Targeted
Turnaround time	Longer	Rapid
Biosafety	Full containment	Enhanced field containment
Logistics	Complex	Self-contained
Military relevance	Strategic confirmation	Tactical decision-making

**Table 3:** Biosafety levels, containment requirements, and laboratory capabilities for viral hemorrhagic fevers.

Biosafety Level	Representative VHFs	Permitted Activities	Key Containment Features	Military Applicability
BSL-2	Dengue, Hantavirus (non-propagative)	Serology, PCR (inactivated)	Class II BSC, PPE	Base hospital labs
BSL-3	CCHFV, Rift Valley fever, inactivated filoviruses	RT-PCR, antigen detection	Negative pressure, HEPA filtration	Deployed/mobile labs
BSL-4	Ebola, Marburg, Lassa	Viral culture, animal studies	Positive-pressure suits, full containment	Strategic reference labs

**Table 4:** Major diagnostic tests, incubation period, and detection windows for viral hemorrhagic fevers.

Virus/Disease	Incubation Period	Diagnostic Method	Target Detected	Specimen	Detection Window	Advantages	Limitations
Ebola virus disease	2–21 days	RT-PCR	Viral RNA	Blood, plasma	Day 1–10 post-symptoms	High sensitivity	Requires high containment
		Antigen RDT	Viral proteins	Blood	Day 2–7	Rapid, field use	Lower sensitivity
		IgM/IgG ELISA	Antibodies	Serum	IgM: day 5–7; IgG: ≥day 10	Surveillance	Late detection
Marburg virus disease	2–21 days	RT-PCR	Viral RNA	Blood	Day 1–10	Gold standard	Limited availability
		ELISA	Antibodies	Serum	≥day 7	Retrospective diagnosis	Not early-stage
Lassa fever	6–21 days	RT-PCR	Viral RNA	Blood	Day 1–7	Sensitive	Genetic variability
		Antigen ELISA	Viral proteins	Serum	Day 2–7	Early detection	Limited access
		IgM/IgG ELISA	Antibodies	Serum	IgM: day 5–7	Epidemiology	Cross-reactivity
Crimean–Congo HF	1–9 days (up to 13)	RT-PCR	Viral RNA	Blood	Day 1–7	High sensitivity	BSL-3 required
		IgM ELISA	Antibodies	Serum	≥day 5	Widely used	Late detection
Dengue fever	4–10 days	RT-PCR	Viral RNA	Serum	Day 1–5	Accurate early diagnosis	Short window
		NS1 antigen	Viral protein	Serum	Day 1–7	Rapid, accessible	Lower sensitivity (secondary infection)
		IgM/IgG ELISA	Antibodies	Serum	IgM: day 4–5; IgG: ≥day 7	Routine use	Cross-reactivity
Yellow fever	3–6 days	RT-PCR	Viral RNA	Blood	Day 1–5	Specific	Narrow window
		ELISA/PRNT	Antibodies	Serum	IgM: day 5; IgG: ≥day 7	Confirmatory	Requires reference labs
Hantavirus infections	1–5 weeks	RT-PCR	Viral RNA	Blood	Early phase (variable)	Confirmatory	Limited routine use
		IgM ELISA	Antibodies	Serum	Early symptomatic phase	Primary test	Cannot detect incubation phase
Rift Valley fever	2–6 days	RT-PCR	Viral RNA	Blood	Day 1–7	Sensitive	Requires containment
		IgM ELISA	Antibodies	Serum	≥day 4–5	Surveillance	Lower early sensitivity

## Data Availability

The original contributions presented in this study are included in the article. Further inquiries can be directed to the corresponding author.

## Abbreviations

The following abbreviations are used in this manuscript:

BMBL	Microbiological and Biomedical Laboratories
BSL	Biosafety Level
CCHFV	Crimean–Congo hemorrhagic fever virus
CDC	Centers for Disease Control and Prevention
CRISPR	Clustered Regularly Interspaced Short Palindromic Repeat
DENV	dengue virus
ELISA	enzyme-linked immunosorbent assays
EVD	Ebola virus disease
GP	glycoprotein
NiV	Nipah Virus
NS1	Non-structural protein 1
NP	nucleoprotein
RT-PCR	Real-time reverse transcription polymerase chain reaction
SFTSV	Severe Fever with Thrombocytopenia Syndrome virus
VHFs	Viral Hemorrhagic Fevers
YFV	yellow fever virus
WHO	World Health Organization
